# A quasi-experiment assessing the six-months effects of a nurse care coordination program on patient care experiences and clinician teamwork in community health centers

**DOI:** 10.1186/s12913-020-4986-0

**Published:** 2020-02-24

**Authors:** Ingrid M. Nembhard, Eugenia Buta, Yuna S. H. Lee, Daren Anderson, Ianita Zlateva, Paul D. Cleary

**Affiliations:** 1 The Wharton School, University of Pennsylvania, Health Care Management Department, 3641 Locust Walk, 207 Colonial Penn Center, Philadelphia, PA 19104 USA; 2 Yale Center for Analytical Sciences (YCAS), 300 George Street, Suite 555, New Haven, CT 06519 USA; 3 Columbia University Mailman School of Public Health, Department of Health Policy & Management, 722 West 168th Street, R476, New York, NY 10032 USA; 4 Weitzman Institute, Community Health Center, Inc., 631 Main St., Middletown, CT 06457 USA; 5 Yale School of Public Health, 60 College St., P.O. Box 208034, New Haven, CT 06520-8034 USA

**Keywords:** Nurse care coordination, Patient care experience, Office visit frequency, Teamwork

## Abstract

**Background:**

Recognition that coordination among healthcare providers is associated with better quality of care and lower costs has increased interest in interventions designed to improve care coordination. One intervention is to add care coordination to nurses’ role in a formal way. Little is known about effects of this approach, which tends to be pursued by small organizations and those in lower-resource settings. We assessed effects of this approach on care experiences of high-risk patients (those most in need of care coordination) and clinician teamwork during the first 6 months of use.

**Methods:**

We conducted a quasi-experimental study using a clustered, controlled pre-post design. Changes in staff and patient experiences at six community health center practice locations that introduced the added-role approach for high-risk patients were compared to changes in six locations without the program in the same health system. In the pre-period (6 months before intervention training) and post-period (about 6 months after intervention launch, following 3 months of training), we surveyed clinical staff (*N* = 171) and program-qualifying patients (3007 pre-period; 2101 post-period, including 113 who were enrolled during the program’s first 6 months). Difference-in-differences models examined study outcomes: patient reports about care experiences and clinician-reported teamwork. We assessed frequency of patient office visits to validate access and implementation, and contextual factors (training, resources, and compatibility with other work) that might explain results.

**Results:**

Patient care experiences across all high-risk patients did not improve significantly (*p* > 0.05). They improved somewhat for program enrollees, 5% above baseline reports (*p* = 0.07). Staff-perceived teamwork did not change significantly (*p* = 0.12). Office visits increased significantly for enrolled patients (*p* < 0.001), affirming program implementation (greater accessing of care). Contextual factors were not reported as problematic, except that 41% of nurses reported incompatibility between care coordination and other job demands. Over 75% of nurses reported adequate training and resources.

**Conclusions:**

There were some positive effects of adding care coordination to nurses’ role within 6 months of implementation, suggesting value in this improvement strategy. Addressing compatibility between coordination and other job demands is important when implementing this approach to coordination.

## Background

Efforts to improve care coordination have increased in recent years due to the recognition that coordination is a central part of high quality care yet is often less than optimal in healthcare [1–4]. Care coordination refers to “the deliberate organization of patient care activities between two or more participants (including the patient) involved in a patient’s care to facilitate the appropriate delivery of healthcare services” ([5], p., 5]). In the United States (U.S.), 35% of patients with serious illnesses or chronic conditions report having experienced a coordination failure [6]. Such failures have resulted in medical complications, preventable hospitalizations, duplicative testing, and morbidity increases [4] estimated to cost the U.S. healthcare system $25 to 45 billion in 2011 [7, 8]. Other countries (e.g. Canada, France, Norway, Sweden, and Switzerland) struggle with care coordination as well, where 30% or more of patients report experiencing coordination failures [6].

To improve care coordination in several countries [4, 9–11], many health systems and organizations have implemented or are implementing nurse care coordination initiatives in which nurses provide additional care and support to patients with coordination needs such as those with a chronic illness, transitioning from hospital to home, or with multiple medical and behavioral health issues [12–14]. In this approach, nurses work closely with designated patients and providers to coordinate multi-specialty care and help patients manage their illnesses. Core responsibilities in this role include monitoring patient health and facilitating development, communication, and delivery of care plans with other care team members [15, 16]. Nurses in many organizations perform these activities, which are within their scope of practice [17, 18]. New programs have structured these activities, clarifying authority, tasks, options, and responsibility, to enhance nurses’ visibility, effectiveness, and efficiency as coordinators. These programs address calls from professional and scientific groups for nurse coordinator roles to be more explicit, developed, and designed deliberately into training and delivery organizations [15, 18–20]. These programs should lead to better experiences for patients and clinicians because assignment of responsibility to one person and coordination improve the logic, continuity, and efficiency of care [5, 12, 18, 21–23].

Currently, two approaches to nurse care coordination are common. In the first, adopted mainly in large health systems and medical groups, a nurse serves exclusively as care coordinator for a panel of patients. This “exclusive-role approach,” has been used, for example, by participants in the U.S. Medicare Care Coordination Demonstration program [24]. In the second “added-role approach”, a nurse performs care coordination in addition to existing responsibilities. Although reviews of research on the first approach indicate mixed results [25–27], increasing evidence from controlled trials indicates that patients with these coordinators experience better technical quality of care, lower hospital readmissions, lower costs, and better care experiences (e.g., provider-patient communication) than patients who do not have a coordinator [12, 17, 28–37].

Little is known about the experiences of patients served by nurses in the added-role approach, which may be pursued more by smaller organizations or those in lower-resource settings, which are many of the settings across the world. There is also limited information, particularly in primary care settings about the effectiveness of this approach, even though these settings are increasingly expected to coordinate care with patients and other providers [38]. In primary care settings such as federally qualified health centers (FQHCs) in the U.S., a type of community health center that serves disproportionately more complex patients with multiple co-morbidities and socioeconomic disadvantages than do private practices and health systems [39], the imperative for coordination is especially great, but there is little evidence about the effects of adding care coordination to the nursing role. Nurses dedicating any increased attention to these tasks may be positive for patients in need and clinicians. On the other hand, the potential positive effects of the added-role approach may not be realized because of the inability to focus exclusively on coordination tasks.

In this manuscript, we examine the early (six-month) effects of a nurse care coordination program in FQHC practices that use the added-role approach for high-risk patients using two measures: care experiences of these patients and clinician-reported teamwork. High-risk patients have complex and/or multiple medical and psychosocial problems, which may require them to see as many as 16 physicians per year, making them most in need of care coordination, most at risk for coordination failures, and most likely to benefit from care coordination, [1, 40] although recent studies suggest that benefits may extend beyond this group [41]. We also examine an indicator of implementation effectiveness, the frequency of patient office visits, and contextual factors because they can influence implementation, and thus program outcomes [42].

We focus on effects in the program’s first 6 months because early experiences with a program are often consequential for long-term success [43–46]. Also, departure from past patterns is often salient to participants early, before they become accustomed to new patterns and adjust expectations, [47] making early assessments a window into program functioning. Currently, there is limited investigation of the early effects of nurse care coordination programs, leaving organizations with little knowledge about what to expect. Research on other patient-nurse and coordination interventions in other settings (e.g., skilled nursing visits in home health care [48–50]) suggests that positive effects can materialize in 6 months.

## Methods

### Study setting and design

This study was conducted in a statewide, multi-site FQHC with 12 sites that provide comprehensive primary medical, dental, and behavioral healthcare services to over 140,000 patients a year. The center serves patients with all types of primary care needs and emphasizes serving the uninsured, underinsured, and special populations such as patients with HIV/AIDS, diabetes, and chronic mental health issues. The FQHC has been recognized as a Primary Care Medical Home by the Joint Commission [51] and a level 3 Patient-Centered Medical Home by the National Commission on Quality Assurance [52]. Thus, each site has demonstrated commitment to patient-centered care, comprehensive care, coordinated care, access to care, and a systems-based approach to quality and safety.

We conducted a cluster quasi-experiment in which pre-post intervention changes in clinician and patient experiences in six sites (clusters) that introduced a nurse care coordination program for high-risk patients using the added-role approach (“intervention group”) were compared to changes in experiences in six sites without the program at the time of our study (“comparison group”). Sites in the comparison group implemented the program after our data collection. The FQHC used a sequential roll-out plan (all locations (3) in one county every 3 months) as it does for certain large-scale initiatives for operational reasons (e.g., maintaining cross-coverage between providers in county and having sufficient resources for implementation). When deciding about comparison sites, the FQHC's leadership selected pairs of sites that were relatively similar based on number of patients, patient population profile, and the organization of sites. Sites were allocated to the intervention group if the intervention could begin sooner there than at pair site, given staff work and training schedules, etc. The selected intervention and comparison sites were similar at baseline and follow-up on all but two characteristics for which we could obtain data (Table 1). Wilcoxon rank-sum tests indicated that the groups differed significantly with respect to percent of patients with Medicare as their health insurer (*p* = 0.02 and *p* = 0.01 at baseline and follow-up, respectively) and percent of patients with “other race” (*p* = 0.05 at baseline). We adjust for these differences in our analyses.

**Table 1:** Comparison of Intervention and Comparison Groups’ Characteristics at Baseline and Follow-up

	Baseline (Median[range])				Follow-up (Median[range])			
Characteristics	Intervention Centers (*N* = 6)	Comparison	Total	*P*-value*	Intervention Centers (*N* = 6)	Comparison Centers (*N* = 6)	Total	*P*-value*
		Centers (*N* = 6)	Centers (*N* = 12)				Centers (*N* = 12)	
Number of patient visits per full-time employee in 6-month period	501 [333;511]	443 [301;528]	450 [301;528]	0.20	918 [631;1114]	841 [611;1000]	841 [611;1114]	0.42
Patient insurance status								
Medicaid patients (%)	71 [59; 74]	64 [55; 81]	68 [55; 81]	0.75	72 [60; 74]	65 [56; 81]	68 [56; 81]	0.94
Medicare patients (%)	8 [4; 10]	13 [9; 17]	10 [4; 17]	0.01	8 [5; 10]	12 [8; 15]	9 [5; 15]	0.02
Private insurance patients (%)	10 [9; 12]	12 [7; 20]	11 [7; 20]	0.42	11 [8; 12]	12 [7; 18]	11 [7; 18]	0.26
Uninsured patients (%)	9 [3; 23]	6 [2; 18]	6 [2; 23]	0.26	9 [3; 22]	6 [2; 16]	7 [2; 22]	0.29
Patient race								
White patients (%)	28 [13; 66]	41 [27; 65]	32 [13; 66]	0.34	27 [13; 64]	38 [25; 63]	31 [13; 64]	0.38
Black patients (%)	8 [7; 20]	15 [2; 22]	12 [2; 22]	0.52	8 [5; 19]	12 [2; 18]	10 [2; 19]	0.87
Hispanic patients (%)	49 [12; 64]	34 [18; 58]	43 [12; 64]	0.26	50 [13; 64]	34 [18; 59]	43 [13; 64]	0.30
Other race patients (%)	4 [2; 5]	5 [4; 12]	4 [2; 12]	0.05	4 [2; 7]	6 [3; 22]	4 [2; 22]	0.20
Race unknown patients (%)	4 [2; 10]	2 [1; 11]	4 [1; 11]	0.19	5 [3; 10]	4 [2; 12]	4 [2; 12]	0.51
Patients eligible for care coordination^	330 [114;1396]	745 [162;1538]	410 [114;1538]	0.63	.	.	.	.
Productivity indicator	1.21 [1.04;1.37]	1.00 [0.96;1.19]	1.12 [0.96;1.37]	0.06	1.23 [1.02;1.28]	1.05 [0.81;1.16]	1.06 [0.81;1.28]	0.07
Supervisor support for staff, indicative of work climate (staff reported, 1–4 scale)~	3.60 [2.96; 3.80]	3.67 [3.49; 3.84]	3.66 [2.96; 3.84]	0.57	3.37 [3.23; 3.88]	3.57 [3.23; 3.93]	3.56 [3.23;3.93]	0.57

^ Baseline values apply to follow-up period as well because the starting sample of eligible patients remained the focus throughout the study. *p-value from Wilcoxon rank-sum tests comparing intervention to comparison centers. ~Supervisor support measured by 5 items from the FQHC’s staff survey

Our primary study outcomes were two indicators of program effectiveness: patient reports about their care experiences and clinician reports of teamwork in their centers. If care coordination programs function as intended, patient experiences, as reflected in responses to questions about care coordination, timeliness of care, and support for self-management should improve, as should clinician teamwork.

Because degree and fidelity of program implementation are critical determinants of program effectiveness, we collected the implementation information that we could, given resource limitations and concerns about staff burden. We obtained information about numbers of telephone calls to patients, but those data turned out to be inconsistent and of poor quality and so are not presented. The other measure of program implementation that we have is the number of patient office visits, which is a proxy measure of accessibility of care, engagement with patients, monitoring, and follow-up to achieve care plan goals (e.g., condition controlled, no preventable hospitalization). If the care coordination program was implemented as intended, there should be an increase in patient office visits in the early months of the program to address outstanding patients’ care needs and self-management training. Research on programs that incorporate the exclusive-role approach has found that primary care office visits increase with coordination programs in the first 2 years, while emergency department visits decline for high utilizers [53]. Over a longer period, not covered by this study, office visits should decline due to better patient health and self-management. Because implementation and effectiveness are often influenced by resources, training, and compatibility with current work, [42, 54, 55] we also assessed these contextual factors via nurse surveys, because these factors may help explain our results. Other non-program specific contextual factors (e.g., employee workload, patient population profile, and supervisor support for workers, which shapes work climate) were examined as well (Table 1).

### Intervention

In intervention sites, every nurse’s role was expanded to include care coordination for adult patients who were expected to benefit most from this effort. These were defined by the organization as patients who were 18 years of age or older, had two or more visits with a primary care provider (PCP) in the past 12 months, and had been identified as “high risk.” Patients were classified as high risk if they had: 1) two or more emergency room visits in the past 12 months; 2) one or more hospitalizations in the past 12 months; 3) a Type 2 diabetes diagnosis on their problem list and a hemoglobin A1C test in the past 12 months greater than 9%; 4) a diagnosis of persistent asthma diagnosis on problem list and two or more asthma control test scores < 19 in the past 12 months; or 5) four or more of specified chronic illnesses on their active problem list, including Type 2 diabetes, chronic obstructive pulmonary disease, hypertension, asthma, coronary artery disease, or behavioral health diagnosis. A subset of the eligible patients (those with greatest immediate need as perceived by staff) was enrolled in the program at the outset due to time and resource constraints. Other patients were also enrolled when a PCP or nurse identified the patient as needing care coordination (e.g., newly discharged from a hospital).

As part of the new program, nurses were expected to work with enrolled patients to help them navigate their healthcare and lead a weekly panel management meeting with enrollees’ PCP and behavioral health provider. The sessions were to be used to review patient progress, identify additional patients who needed coordination, and plan coordinated care. To implement the program, the organization introduced the nurse care coordinator role to all staff via meetings and other communications (e.g., newsletters). It also provided three resources to nurses to support their effectiveness as coordinators: training, a “playbook”, and an electronic dashboard. All nurses in the intervention sites received 23 h of training over a period of 2 to 3 months from experts within the organization and outside consultants. The training covered care plan development, panel management, documentation, transition care support, motivational interviewing, self-management goal setting, chronic disease management, and behavioral health disorders — evidence-based components of nurse care coordination [17]. The playbook provided instructions for each task within the new nurse role, information on additional resources, and measures to evaluate performance. The electronic dashboard leveraged information in the organization’s electronic health record system, which aided nurse tracking of patients and activities. No other group was assigned care coordination responsibilities. The organization reinforced its commitment to the role change by monitoring nurse performance and providing feedback reports to nurses. It was expected that the program would lead to more coordinated and timely care, greater patient support for self-management, and care for mental health.

### Study outcomes

#### Patient care experiences

We collected patient surveys that asked about care experiences during two periods at each center. The first (baseline) period covered the 6 months prior to start of nurse training in the intervention centers, and was before nurses were told about the intervention and patients who would be in the program were known. In intervention and comparison centers, we invited a random sample of the high-risk (i.e., program-eligible) patients described earlier that had visited the center in the preceding 6 months (*N* = 5525) to complete the Consumer Assessment of Healthcare Providers and Systems Clinician & Group (CG-CAHPS) survey [56, 57] and Patient-Centered Medical Home (PCMH) Supplemental Item Set [57, 58]. These surveys assess multiple aspects of patient care experiences, [57, 59] and have been used in other studies of care coordination [60, 61]. The sites already administered these surveys for performance monitoring. With funding provided by the CAHPS Program, we supplemented sites’ surveying to capture the patients in this study.

We assessed the program’s impact using patients’ responses to questions about four aspects of care targeted by the program and therefore expected to be affected by experiencing the program: timeliness of care, coordination of care, support for patient self-management, and care for mental health. Timeliness of care was hypothesized to increase because patients in the program would have priority access to care; their nurse care coordinators would try to be highly responsive. Coordination of care for program enrollees was to improve because nurses would focus on ensuring that enrollees’ needs were met as seamlessly as possible. Support for self-management and care for mental health were additional program foci and areas of training for nurses; therefore, we expected that nurse efforts in these areas would be reflected in patient reports of their experiences. We focused on these four standard measures of patient care experience, rather than care coordination alone, recognizing that nurse care coordination efforts should manifest in multiple ways [19, 20]. Table 2, Part A lists the items used from the CG-CAHPS survey to measure these aspects of care, response options, and the reliability of the scales in our sample. Patients indicated whether they experienced the action described in each question using a four-point scale (1 = never to 4 = always) or No (=1)/Yes (=4) response. We averaged responses for the items in each composite to arrive at a score for each aspect of their experience. The four composite scores are highly correlated (*p*-values < 0.001), so to simplify analyses and presentation, we averaged them to arrive at an overall patient care experience score for each person.

**Table 2:** Study Measures

**A. Patient-reported care experience (4 components)**	
**Timeliness of care** (Cronbach’s alpha (α) = 0.89)	
▪ When you phoned this provider’s office to get an appointment for care you needed right away, how often did you get an appointment as soon as you needed?	
▪ When you made an appointment for a check-up or routine care with this provider, how often did you get an appointment as soon as you needed?	
▪ How often were you able to get the care you needed from this provider’s office during evenings, weekends, or holidays?	
▪ When you phoned this provider’s office during regular office hours, how often did you get an answer to your medical question that same day?	
▪ When you phoned this provider’s office after regular office hours, how often did you get an answer to your medical question as soon as you needed?	
▪ How often did you see this provider within 15 min of your appointment time?	
**Care coordination** (Cronbach’s alpha (α) = 0.73)	
▪ How often did this provider seem to know the important information about your medical history?	
▪ When this provider ordered a blood test, x-ray, or other test for you, how often did someone from this provider’s office follow up to give you those results?	
▪ Did you get the help you needed from this provider’s office to manage these different providers and services?	
▪ How often did the provider named seem informed and up-to-date about the care you got from specialists?	
▪ How often did you and anyone in this provider’s office talk about all the prescription medicines you were taking?	
**Support for patient self-management** (Cronbach’s alpha (α) = 0.65)	
▪ In the last 6 months, did anyone in this provider’s office talk with you about specific goals for your health?	
▪ In the last 6 months, did anyone in this provider’s office ask you if there are things that make it hard for you to take care of your health?	
**Care for mental health** (Cronbach’s alpha (α) = 0.78)	
▪ In the last 6 months, did anyone in this provider’s office ask you if there was a period of time when you felt sad, empty or depressed?	
▪ In the last 6 months, did you and anyone in this provider’s office talk about things in your life that worry you or cause you stress?	
▪ In the last 6 months, did you and anyone in this provider’s office talk about a personal problem, family problem, alcohol use, drug use, or a mental or emotional illness?	
**B. Clinician-reported teamwork (2 components)**	
**Interprofessional Collaboration** (Cronbach’s alpha (α) = 0.77)	
▪ Nurses and physicians plan together to make decisions about care for complex patients.	
▪ Open communication between care providers takes place as decisions are made for complex patients.	
▪ Decision-making about patient care for complex patients is well-coordinated.	
▪ The input of ancillary staff is regularly sought when developing care plans.	
**Relational coordination** (Cronbach’s alpha (α) = 0.75)	
▪ The people on this team share my goals for the care of patients.	
▪ The people on this team know about the work I do with patients.	
▪ The people on this team respect me and the work I do with patients.	
▪ The people on this team communicate with me in a timely way about the status of patients.	

Note: Cronbach’s alpha (α) above 0.70 indicates satisfactory reliability of a measure and between 0.50 and 0.70 indicates moderate reliability. The reported alphas are based on baseline data. For the first two aspects of care, patients indicated whether they experienced the action described in each question using a four-point scale (1 = never to 4 = always), except for the third item in the care coordination scale for which they replied No (=1) or Yes (=4). For the third and fourth aspects of care, they replied No (=1) or Yes (=4). For staff-reported teamwork, staff responded using a four-point response scale (1 = strongly disagree to 4 = strongly agree)

After the program had been in effect for 6 months following nurse training, we again invited a random sample of program-eligible patients that had visited the center in the preceding 6 months (*N* = 4661) to complete the CG-CAHPS survey with additional items. All 145 program enrollees received an invitation by design. Follow-up at 6 months allowed us to avoid contamination of the comparison group: per the organization’s fixed roll-out plan, the program (training) was scheduled to begin in the first set of comparison centers at this time. This planned endpoint also aligned with our study objective to assess early effects of the added-role approach.

In both the baseline and follow-up periods, we mailed a copy of the survey in English and Spanish to each patient in the sample. Approximately 2 weeks after the first mailing, members of the sample were sent a thank you/reminder postcard. Approximately 2 weeks after that, another survey package was mailed to those who had not responded. If no response was received after two to three more weeks, we called the patients. A minimum of six calls per person were made on different days and at different times of the week.

In the baseline period, 3209 patients of the 5525 contacted (58%) answered the survey; of those, 3007 (94%) confirmed having visited the center in the prior 6 months (intervention group = 934; comparison group = 2073). In the follow-up period, 2306 patients of the 4661 contacted (49%) answered the survey; of those, 2101 (91%) confirmed having visited the center in the prior 6 months (intervention group sample size = 774; comparison group sample size = 1327). In total, 943 patients answered the survey in both periods (643 in control group; 300 in intervention group), and 113 program enrollees responded (78% of the 145 enrolled).

#### Teamwork

During the month in which we began both the baseline and follow-up patient surveys, we administered an “organizational assessment survey” via the internet or paper to all primary care team members (PCP, nurses, medical assistants, and behavioral health providers). We recruited team members to participate via informational presentations during lunchtime staff meetings and email, and confirmed willingness to participate via signed consent forms. The survey consisted of validated survey scales for assessing core aspects of teamwork i.e., relational coordination and interprofessional collaboration [62–64]. Interprofessional collaboration refers to the degree of cooperation among individuals with different disciplinary backgrounds [65], while relational coordination refers to the presence of high-quality communication and relationships characterized by shared goals, shared knowledge, and mutual respect needed for task integration [66]. Each scale included four items (Table 2, Part B). Team members indicated their level of agreement with each item using a four-point response scale (1 = strongly disagree to 4 = strongly agree). Because scores for the two scales were highly correlated (*p* < 0.001), we averaged them to arrive at a summary teamwork score reported by each respondent.

At baseline, 96 of 190 (51%) team members completed the survey (intervention group = 43; control group = 53). At follow-up, 135 of 188 (72%) members completed the survey (intervention group = 57; control group = 78). Sixty members participated at both baseline and follow-up. We used their responses in our analyses to assess program effect based on the experiences of a stable population and minimize the possible confounding effect of respondents new to the centers. This longitudinal sample was 39% PCPs, 22% nurses, 24% medical assistants, and 15% behavioral health providers. The majority were female (71%), full-time staff (89%), and with the organization more than 2 years (82%). Except for the percentage with more than 2 years with the organization (63%), this sample was demographically like the full sample consisting of 33% PCPs, 23% nurses, 28% medical assistants, 18% behavioral health providers, 83% female, and 88% full-time staff.

### Implementation measures

#### Office visit frequency

We obtained information about patients’ number of office visits via response to a question in the CG-CAHPS survey: “In the last 6 months, how many times did you visit this provider to get care for yourself?” Seven response options were offered: none, 1 time (coded as 1), 2 (coded as 2), 3 (coded as 3), 4 (coded as 4), 5 to 9 (coded as 7, the midpoint), and 10 or more times (coded as 10). Patients who did not recall any visits were excluded from study (*N* = 202 (7%) at baseline and 205 (9%) at follow-up).

#### Contextual factors: training, resources, and compatibility with other job demands

The organizational survey administered to primary care team members during the follow-up period included additional questions for nurses about program training, resources, and their new role’s compatibility with other job demands, which we used to assess whether these factors posed a challenge to implementation and effectiveness. Four items were adapted from Venkatesh et al.’s [67] facilitating attributes scale: “I have the resources necessary to coordinate care for complex patients,” “I have the knowledge necessary to coordinate care for complex patients,” “Coordinating care for complex patients is not compatible with other tasks that I’m required to perform,” and “It is easy for me to coordinate care for complex patients.” A fifth resource-related item drew from the FHQC’s employee survey: “I have adequate authority to carry out my work.” We asked nurses at intervention centers to report their level of agreement with each statement (1 = strongly disagree to 4 = strongly agree). Other non-program specific contextual factors that can affect implementation (e.g., supervisor support for workers and workload) and could be assessed for intervention and comparison groups at baseline and follow-up were evaluated for potential inclusion as covariates.

### Covariates

In models assessing patient care experience (study outcome) and office visit frequency (implementation indicator), we included person-level characteristics that have been shown to be related to reports about healthcare experiences: age, gender, education, race/ethnicity, overall health status, and mental health status [68]. These were all collected via the CG-CAHPS survey, measured as categorical variables (see Table 3 for categories), and treated as time-varying covariates because some could change from baseline to follow-up. In models assessing teamwork, we included person-level characteristics, obtained via the staff survey, that may influence interactions: profession, gender, full-time status, and years in the organization. In all models, we included characteristics that differed between intervention and comparison centers: percent of patients with Medicare and other race. Characteristics that did not differ were not included.

**Table 3:** Description of the Patient Sample by Time Period and Affiliated Center’s Intervention Status

	Baseline (*N* = 3007)			Follow-up (*N* = 2101)		
	Intervention Centers	Comparison Centers	p-value*	Intervention Centers	Comparison Centers	p-value*
	(*N* = 934)	(*N* = 2073)		(*N* = 774)	(1327)	
	n(%)	n(%)		n(%)	n (%)	
Age Group			0.01			0.04
18 to 24	21 (2.25%)	36 (1.74%)		16 (2.07%)	15 (1.13%)	
25 to 34	73 (7.82%)	166 (8.01%)		72 (9.30%)	85 (6.41%)	
35 to 44	157 (16.8%)	268 (12.9%)		115 (14.9%)	167 (12.6%)	
45 to 54	293 (31.4%)	632 (30.5%)		205 (26.5%)	389 (29.3%)	
55 to 64	255 (27.3%)	624 (30.1%)		234 (30.2%)	400 (30.1%)	
65 to 74	86 (9.21%)	251 (12.1%)		90 (11.6%)	186 (14.0%)	
75+ years	36 (3.85%)	84 (4.05%)		33 (4.26%)	64 (4.82%)	
Missing	13 (1.39%)	12 (0.58%)		9 (1.16%)	21 (1.58%)	
**Gender**			0.34			0.07
Female	586 (62.7%)	1261 (60.8%)		504 (65.1%)	810 (61.0%)	
Male	348 (37.3%)	812 (39.2%)		270 (34.9%)	517 (39.0%)	
Missing						
Highest Grade Completed			< 0.001			0.002
< = 8th grade	150 (16.1%)	199 (9.60%)		130 (16.8%)	144 (10.9%)	
Some high school	166 (17.8%)	386 (18.6%)		174 (22.5%)	270 (20.3%)	
High school grad or GED	295 (31.6%)	762 (36.8%)		228 (29.5%)	470 (35.4%)	
Some college/2-yr degree	225 (24.1%)	515 (24.8%)		174 (22.5%)	330 (24.9%)	
4-year college graduate	43 (4.60%)	110 (5.31%)		32 (4.13%)	51 (3.84%)	
More than 4-year college	25 (2.68%)	70 (3.38%)		16 (2.07%)	23 (1.73%)	
Missing	30 (3.21%)	31 (1.50%)		20 (2.58%)	39 (2.94%)	
Race/Ethnicity			< 0.001			< 0.001
White	305 (32.7%)	979 (47.2%)		252 (32.6%)	612 (46.1%)	
Hispanic	478 (51.2%)	607 (29.3%)		392 (50.6%)	400 (30.1%)	
Black	68 (7.28%)	239 (11.5%)		75 (9.69%)	162 (12.2%)	
Asian	10 (1.07%)	38 (1.83%)		6 (0.78%)	21 (1.58%)	
Other	36 (3.85%)	107 (5.16%)		38 (4.91%)	104 (7.84%)	
Missing	37 (3.96%)	103 (4.97%)		11 (1.42%)	28 (2.11%)	
Overall Health Status			0.07			0.49
Excellent	61 (6.53%)	146 (7.04%)		47 (6.07%)	109 (8.21%)	
Very Good	111 (11.9%)	274 (13.2%)		78 (10.1%)	146 (11.0%)	
Good	260 (27.8%)	652 (31.5%)		230 (29.7%)	383 (28.9%)	
Fair	351 (37.6%)	721 (34.8%)		291 (37.6%)	478 (36.0%)	
Poor	137 (14.7%)	263 (12.7%)		117 (15.1%)	188 (14.2%)	
Missing	14 (1.50%)	17 (0.82%)		11 (1.42%)	23 (1.73%)	
Mental Health Status			0.06			0.20
Excellent	119 (12.7%)	287 (13.8%)		100 (12.9%)	168 (12.7%)	
Very Good	144 (15.4%)	352 (17.0%)		107 (13.8%)	234 (17.6%)	
Good	253 (27.1%)	594 (28.7%)		222 (28.7%)	361 (27.2%)	
Fair	303 (32.4%)	603 (29.1%)		253 (32.7%)	393 (29.6%)	
Poor	97 (10.4%)	219 (10.6%)		81 (10.5%)	144 (10.9%)	
Missing	18 (1.93%)	18 (0.87%)		11 (1.42%)	27 (2.03%)	

* *p*-values from chi-square test comparing patients in intervention versus comparison centers, without adjustment for clustering

### Analyses

We conducted two analyses of patient care experience. First, we used a differences-in-differences approach to assess whether the difference in experiences between baseline and follow-up in intervention centers differed significantly from the corresponding difference observed in comparison centers, using data from all survey respondents. This intent-to-treat approach assesses whether the program affected care for all program-qualifying patients at intervention sites, not just enrollees. We used separate mixed linear, also termed multi-level, regression models that included fixed effects for intervention group status (0 = comparison group, 1 = intervention group), period (0 = baseline, 1 = follow-up), intervention-by-period interaction, and covariates. We also included random effects for person, PCP, and center to account for clustering and correlation between repeated measures of the same person, between persons affiliated with the same PCP care team, and between PCP care teams within the same center, respectively. In the models, we focused on the intervention-by-time interaction term, which indicate whether the change over time in the intervention centers was greater than in comparison centers (i.e., whether there was a significant program-intervention effect). Our second set of analyses compared the experiences of only patients enrolled in the coordination program at intervention sites (*N* = 95 with complete data of the 113 enrolled) to the experiences of eligible patients at comparison sites. We included the baseline data from all surveyed patients because all were program-eligible. This focused, sub-group analysis assessed the effect of the program on those treated.

For our analysis of teamwork, we again used mixed linear regression models that accounted for clustering within clinics and included fixed effects for intervention group status, period, intervention-by-period interaction, and covariates. We analyzed data from clinicians who had complete data in both the baseline and follow-up surveys as noted above, and again studied difference-in-differences. We used this same statistical technique to assess changes in office visit frequency, our implementation indicator. We present adjusted least squares (LS) means with associated standard errors for our measures.

For our analysis of contextual factors, we calculated the percentage of nurse respondents (*N* = 27) who agreed or strongly agreed (response of 3 or 4) with each of the statements in the contextual factors measure. We regarded percentages of 75% or greater as indicative that the factor did not substantially undermine implementation and outcomes.

## Results

Table 3 presents participating patients’ characteristics at baseline and follow-up for intervention and comparison centers. Intervention center patients were younger, less educated, and less likely to be White (*p* < .05), but did not differ with respect to gender and health status.

Our first analysis of patient care experiences, using data from all survey respondents, indicated that there was not a statistically significant difference in changes between intervention and comparison groups (*p* > 0.10). In other words, the program did not change the experience of patients at intervention centers as a whole significantly. Our second analysis focused on program enrollees, however, showed modest improvement in care experiences for this subgroup (*p* = 0.07).

**Fig. 1:**
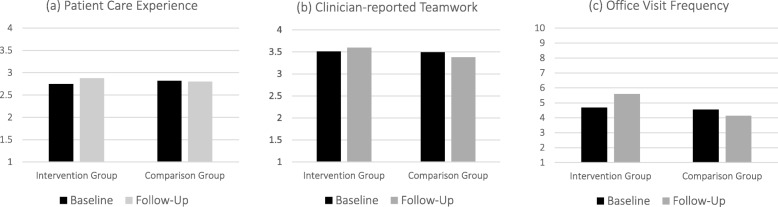
Patient Experience, Clinician-reported Teamwork and Office Visit Frequency for Intervention and Comparison Groups

Table 4 shows the results of the focused analysis, as well as our analysis of changes in clinician teamwork and patient office visit frequency, our program implementation indicator. The table presents the adjusted LS-means for each measure in intervention and comparison groups at baseline and follow-up and for the difference in change from baseline to follow-up between the two groups (difference-in-difference). Row 1 shows that patient care experience scores increased in the intervention group (2.75 to 2.88, 5%), while scores in the comparison group remained about the same (with slight decrease from 2.82 to 2.80) (*p* = 0.07). Row 2 shows that clinician-reported teamwork increased in the intervention group (3.51 to 3.60, 3%) and decreased in the comparison group (3.49 to 3.38, 3%), however, the difference was not significant (*p* = 0.12). Row 3 shows that an increase in office visit frequency, our implementation indicator, occurred in the intervention group (for patients enrolled in the program), while a decrease in office visits occurred in the comparison group. Patients enrolled in the program had 1.33 more visits than those in the comparison group, a significant difference (*p* < 0.001). Figure 1 graphically presents the results for each measure.

**Table 4:** Effect of Program on Patient Care Experiences, Clinician-reported Teamwork, and Office Visit Frequency

Outcome	N	Intervention				Comparison				Difference in change from baseline to follow-up (F-B) for intervention vs. comparison (difference in difference)		
		Baseline (B)		Follow-up (F)		Baseline (B)		Follow-up (F)				
		LS-mean	SE	LS-mean	SE	LS-mean	SE	LS-mean	SE	LS-mean	SE	p
1. Patient care experience	3638	2.75	0.07	2.88	0.10	2.82	0.04	2.80	0.04	0.15	0.08	0.07
2. Clinician teamwork	60	3.51	0.12	3.60	0.13	3.49	0.11	3.38	0.12	0.21	0.13	0.12
Implementation Indicator												
3. Office visit frequency	3638	4.69	0.20	5.60	0.34	4.55	0.14	4.13	0.14	1.33	0.32	< 0.001

Notes: Results presented are for analysis comparing effects for program enrollees. *N* = number subjects analyzed, which is less than total patients in Table 3 because some patients participated in both periods or had missing data; LS-mean = least squares mean (mean adjusted for covariates). The scale ranged from 1 to 4 for patient care experience and staff teamwork, and from 1 to 10 for office visit frequency. Higher values are better

Most nurse respondents agreed or strongly agreed that training and resources were adequate for their role as care coordinators: 75% (standard error (S.E.) = 0.09) for having the resources necessary; 87% (S.E. = 0.10) for having the knowledge necessary; and 79% (S.E. = 0.10) for having adequate authority to perform the work required – all theorized prerequisites for the coordination program to have desired effects. On the other hand, 41% of nurses (S.E. = 0.08) agreed or strongly agreed that “Coordinating care for complex patients is not compatible with other tasks that I’m required to perform, and only 25% (S.E. = 0.06) that “It is easy for me to coordinate care for complex patients.”

## Discussion

The results of our quasi-experimental study of the effect of implementing a nurse care coordination program in community health centers suggest that adding care coordination to the nursing role is associated with improvement for program enrollees in key program goals, including greater patient access to and engagement with healthcare providers (as indicated by office visit frequency, our implementation indicator) and better patient care experiences ranging from care coordination to care for mental health (key outcome). Moreover, our results indicate that these positive patient effects can occur in a relatively short period following program implementation (6 months), whereas improved clinician teamwork and spillover effects to all high-risk patients may require time. To our knowledge this is the first multi-center study to evaluate the effect of the added-role approach to nurse care coordination on patients’ and staff’s experiences, the early effects, and in community health centers, a setting in which care coordination has been under-studied despite its importance for the vulnerable populations such centers serve. The experience in these low-resource health centers may be informative for other low-resource settings across the world that seek to improve care coordination and patient care experiences. It may also be informative for developed health systems with greater resources as a review of 18 systematic reviews of nurse care coordination in primary care showed uncertainty about the best approach to this role in developed countries (comparable to Australia) as well [10].

The positive although modest trajectory of effects for program enrollees, particularly in a short timeframe, is notable because uncertainty about the effectiveness of the added-role approach to nurse coordination in particular has prevailed. The question of whether the exclusive-role approach is the only way to garner the benefits of nurse coordination lingered. This study contributes to the literature on care coordination by providing evidence that supports the added-role approach as a potential alternative, if compatibility with other job demands can be cultivated. The results indicate that the effects of this approach, with training and resources provided, are comparable in some respects to larger programs focused on improved care management for high-risk patients. The two-year analysis of the Comprehensive Primary Care Initiative (CPCI) — launched by the U.S. Centers for Medicare and Medicaid Services and 39 other payers and which also used the CG-CAHPS survey to study patient care experience — similarly found a significant but small positive effect on patient self-management [60]. The more-intensive CPCI required participating primary care practices to build their capacity for care coordination and other skills (e.g., patient engagement) and supported practices by providing them with enhanced payment, data feedback and learning resources [60]. The CPCI had no significant effect on other aspects of patient care experiences also studied here, including care coordination and timeliness of care.

The results for the added-role approach however mirror larger programs with respect to primary care office visits. A three-percent increase in primary care visits occurred in the first 2 years of the CPCI as well (compared to 5 % here), [60] and the number of visits increased by 1.3 (same as in this study) in the first six-months of Bridges to Care (B2C)—a hospital-initiated, community-based program [53]. The consistency in effects across programs is notable because our study differed in population (all adults versus adults ages 65 and older) and setting (community health centers versus primary care practices and hospitals). Although extended study is needed for assessing the degree and persistence of effects found here and therefore to be pursued in future research, these early results are valuable for understanding the trajectory of effects. Moreover, our findings add to research that has found significant effects on care of implementing other types of nursing interventions (e.g., skilled nursing visits in home healthcare) in 6 months [48–50, 53].

Greater, short-term improvement in patient care experiences with the added-role approach than we found may be possible under other circumstances. In the centers studied, although leadership made sizable investments in training and infrastructure to support the program, financial constraints limited their ability to provide nurses with extended, protected time for care coordination activities, not unusual in lower-resource settings. Thus, many nurses felt that there was incompatibility between their other job demands and care coordination. Incompatibility can exist because coordination has many components as described in the Intervention section and requires time (e.g., to speak with patients and providers, to schedule care, etc.). When a nurse is focused on coordination tasks for high-risk patients, she or he must reduce the time spent on tasks for other patients, a tradeoff perceived as incompatibility between roles by many nurses. Operations research shows that slack time and flexibility in task timing can be critical for new initiatives, particularly in the early stage of implementation involving role redesign [69–71]. Larger effects of the added-role approach may emerge if additional time is allotted for coordination activities. The study organization chose the added-role over the exclusive-role approach because nurses felt that non-coordinating nurses would be left with less stimulating work and interactions (e.g., immunizations) and their skills would decline; all nurses wanted involvement in improving care for their neediest patients. An optimal level of involvement in care coordination may require shifting some tasks to other team members (e.g., having medical assistants monitor the list of patients for care coordination) [72]. With relief from some tasks, allowing nurses more time for care coordination, the added-role approach may be an even better solution for patients and nurses. Thus, our results imply that organizations and health systems using this solution will need to provide protected time for care coordination activities, which may mean changing other workers’ roles. When roles change, it is important that representatives of all affected roles participate in implementation planning to increase consideration of how job components interact and facilitate adjustments in job demands to increase compatibility among roles and thus effectiveness [73].

There are several possible reasons why clinician teamwork did not increase significantly during the study. One possibility is that more time was needed to improve teamwork. Organizational research on teams has found that teamwork—which involves not only behavioral processes (e.g., collaboration and coordination) but also emergent states that support those processes (e.g., mutual respect and psychological safety that taking interpersonal risks such as asking questions will not be held against you) [63, 74]—takes extended time to develop and solidify, [75, 76] particularly when compounded by role changes. A literature review found that teams often pass through development phases and try variations in the way they work before they settle into an equilibrated role structure, especially when new members or roles are introduced [77]. A second possible explanation for the non-significant change is that care coordination is largely a nurse-patient intervention versus a nurse-other clinician intervention in the early phase because nurses work to understand patient circumstances and needs first. A study of non-licensed care coordinators embedded into primary care practices found that these coordinators did not change existing care team functioning, yet patients reported significantly better experiences from these coordinators’ efforts to improve largely nonmedical tasks [78], which suggests that the coordinator-patient interaction is most central and salient. A third possibility is a ceiling effect: Teamwork was already high (above 3 on the 4-point scale) in all centers, leaving limited room for improvement on the scale. Other potential reasons beyond the three presented here are possible. Ethnographic studies involving direct observation of care teams, interviews of care teams and patients, and sampling of centers for greater variation in teamwork scores are needed to examine the possibilities and better understand changes in teamwork following implementation of nurse care coordination.

We assessed early program impact in part because of project imitations, but also because early effects can be consequential for long-term success [43–46]: assessment of early effects allows for managerial intervention if needed to improve program adoption and impact. In addition to the managerial intervention needed to address compatibility between care coordination and other job demands (e.g., implementing protected time and task-shifting between team members), our findings point to the need for managers to plan for an increase in office visits associated with care coordination in the early months (and potentially years). Extended office hours during the week, weekend appointments, and/or additional staffing during existing work hours are potential solutions for increasing capacity to accommodate additional office visits. If funds are not available for these solutions, organizations will need to consider ways to increase efficiency with existing resources to create more time for office visits (e.g., using group visits, electronic communication, etc.). Office visits, especially during the early part of the program, are important because they provide the opportunity for care teams and patients to build rapport and partnership, understand and address care needs that are often complex (requiring physical assessments and conversation), and train patients in self-management. These interactions are key for promoting the “deliberate organization of patient care activities…to facilitate appropriate delivery”, the definition of care coordination” ([5], p., 5). Not planning for increased visits, alongside training, resources, and compatibility of care coordination with other nurse-job demands is likely to minimize the potential for the added-role approach to build on early progress to produce greater and sustained gains long term.

There are several limitations to our study. First, our results are based on the experiences of patients and staff in a small sample of centers affiliated with one organization in one state. Thus, our results may not generalize to other settings. Second, we were not able to randomize centers to the intervention and control groups. This could result in biased results, although our quasi-experimental design and the similarity in intervention and comparison groups’ characteristics should provide valid insights. Additionally, we adjusted for two observed patient population differences between intervention and comparison sites, and for key patient characteristics. Third, selection bias may have affected our results. Our participation and response rates, however, are comparable or better than other studies of low-income patients [69, 79] and clinicians [80]. Still, it is possible that those who selected to participate differed from those who did not. The latter might report better or worse experiences than participants, which might strengthen or dampen found effects. However, given the modest effects found across the greater than majority of the study population, conclusions likely hold. Finally, we did not assess the full range of experiences that might have been affected (e.g., nurse helpfulness), focusing instead on core measures of patient care experience [58].

## Conclusion

Poor care coordination is a pervasive problem that affects millions of people [6, 81] and has been slow to improve, with fewer than half of coordination metrics in the U.S., for example, having improved since 2001 [4]. Our findings suggest that adding care coordination to the nursing role can spur some improvement in a relatively short time in key metrics, specifically, patient care experiences and accessing care via office visit frequency. With more time, improvement in clinician interactions–from their perspective̶–may be substantial as well. However, compatibility between existing nurse job demands and care coordination needs to be addressed to realize greater benefit from this approach.

## Data Availability

The datasets generated and analysed during the current study are available from the corresponding author on reasonable request.

## Abbreviations

AHRQ: Agency for Healthcare Research and Quality
CG-CAHPS: Consumer Assessment of Healthcare Providers and Systems Clinician & Group Survey
FQHC: Federally Qualified Health Center
